# Open Access Estimation of deltamethrin residues in cow’s and goat’s environment and trials to reduce its level in milk

**DOI:** 10.14202/vetworld.2018.606-611

**Published:** 2018-05-11

**Authors:** Halla E. K. El Bahgy, Hend A. Elbarbary, Samar S. Ibrahim

**Affiliations:** 1 Hygiene and Veterinary Care, Department, Faculty of Veterinary Medicine, Benha University, 13736 Moshtohor, Toukh, Qalyubia, Egypt; 2 Department of Food Hygiene, Faculty of Veterinary Medicine, Benha University, 13736 Moshtohor, Toukh, Qalyubia, Egypt; 3 Department of Forensic Medicine and Toxicology, Faculty of Veterinary Medicine, Benha University, 13736 Moshtohor, Toukh, Qalyubia, Egypt

**Keywords:** deltamethrin, freezing, high-performance liquid chromatography, microwaving treatment, milk, pesticides residues

## Abstract

**Aim:**

The present study was aimed to estimate deltamethrin residues in cow’s and goat’s environment over a certain period of time post-application, to identify the role of both feed and water as a source of pesticides, and to conduct some trials to reduce their levels in milk.

**Materials and Methods:**

A total of 80 water and feed samples (40 of each) and 120 milk samples (80 cow’s milk and 40 goat’s milk) were collected. Fresh milk samples were collected directly from the udder as well as from feed and water before application and 1^st^, 2^nd^, 3^rd^, 7^th^, 15^th^, 21^st^, and 35^th^ days after insecticide application.

**Results:**

Deltamethrin residues were detected after its application in both water and feed at different levels up to the first 3 days and in all cow’s and goat’s milk samples at 35^th^ day. The highest levels were detected in milk samples at the 2^nd^ day then at the 7^th^ day followed at the 15^th^ day after application as such levels were above the maximum residual limits. By microwaving the polluted cow’s milk samples, deltamethrin residues were not detected without influencing the chemical composition of the milk. However, on freezing of milk, the deltamethrin residues reached 12.6±3.24 μg/L in association with a significant decline in the concentration of fat.

**Conclusion:**

Microwaving of milk is an effective method to decline deltamethrin concentration in milk.

## Introduction

Deltamethrin is a synthetic type II pyrethroids insecticide [1]. It is one of the most known potent insecticides, and it is largely used in veterinary medicine as acaricide against animal infestations as well as agriculture formulations to control numerous insect pests on fruits, vegetables, and field crops [2].

Deltamethrin is derived from natural pyrethrins (esters of chrysanthemic and pyrethric acid extracted from chrysanthemum flowers, *Chrysanthemum cinerariaefolium*, and related species). It is insoluble in the water, soluble in acetone, dimethyl sulfoxide, N,N-dimethylformamide, benzene, xylene, cyclohexanone, and ethyl acetone, and slightly soluble in ethanol and isopropanol. It is stable in the acidic and neutral solutions and at 40°C in the dark and at room temperature in the light. However, it is unstable in alkaline solutions [3].

The major toxic effects of deltamethrin include choreoathetosis, hyperexcitability, and salivation [4]. These effects are generally rapid in onset and brief in duration [5]. Moreover, there is a reported case of a 30-year-old male who died within 2 days after consuming about 30 mL of deltamethrin. Another possible adverse effect of deltamethrin is teratogenesis [6].

Egypt is one of the largest users of pesticides to duplicate its animal and plant production power. The monitoring of pesticide residues is very important for controlling the safety of milk and dairy products consumed by infants, children, and adults throughout the world. Especially, milk is known as a nutritious, wholesome food consumed globally and it is an inexpensive source of protein and calcium essential for promoting the growth of children and the general good health of the population [7].

Nowadays, there is a great concern about the quality of the food consumed by human beings in the modern world. In addition, pesticide residues cause many troubles in food [8]. For example, insecticides affect the activity of starter culture and the quality of the dairy products as they elongate the coagulation time in cheese with the formation of many holes [9].

Countries have enacted regulations to set the maximum residue level (MRL) of such pesticide residues in milk and dairy product to protect the consumers’ health [10]. This is guided by different analytical approaches such as thin-layer chromatography, liquid chromatography, and immunochemistry [11]. However, the most important approach is based on high-performance liquid chromatography method (HPLC) [12,13].

Insight of these facts, the current study aimed to estimate deltamethrin residues in cow’s and goat’s environment over a period of time post-application, to identify the role of both feed and water as a source of pesticides, and to apply some trials for reduction of its levels in milk.

## Materials and Methods

### Ethical approval

The study was approved by the Research Committee of the Faculty of Veterinary Medicine, Benha University, Qualyobia, Egypt.

### Animals

Two farms, cow and goat ones, were included in this study located at Qalyubia Governorate. The experiment was carried out on apparent healthy lactating cows (n=10) and goats (n=5). Each animal was sprayed by the same person with a single therapeutic concentration of the deltamethrin (Butox^®^ 50 with active ingredient deltamethrin 50 mg/L) with recommended quantity (50 mL/100 L water) on the back from the shoulder to sacrum under field condition.

### Feed and water samples

Eighty water and feed samples were collected (n=5 of each group for water and n=5 of each group for feed) at different intervals before and after the application of deltamethrin. Feed and water samples were 500 mL and 200 g respectively. Water samples were collected in glass bottles, then immediately were filtered through a 0.7 µm glass filter, and were stored in darkness at 4°C. While feed samples (Corn-Drees-Silage-Soya-Bran-Linseed-Molasses-Hey-Grille feed-Berseem) were collected from feed troughs. The feed samples were stored at −20°C until processing.

### Milk samples’ collection

A total of 120 milk samples (80 cow’s milk and 40 goat’s milk) were collected at different intervals at zero time, 1^st^, 2^nd^, 3^rd^, 7^th^, 15^th^, 21^st^, and 35^th^ days after application and in addition to control group. The milk samples were collected in sterilized 50 ml Falcon tubes. They were immediately transferred to the laboratory for examination.

### Treatments of polluted milk samples to control their deltamethrin residues

In Egypt, there is no interest for the consumer to drink goat’s milk due to its pronounced flavor. Hence, focusing on treatment of cow’s milk is necessary.

10 cow’s milk samples were collected at the 2^nd^ day after insecticides application (highest deltamethrin concentration) and divided into two parts. The first part was treated by microwave at 900 W for 75 s according to Tremonte et al. [14], while the second part was treated by freezing at −20°C for 1 week after collection [15].

### Chemical analysis of milk composition

Fresh and treated milk samples (microwaving or freezing) were analyzed for fat percentage, solid-not-fat percentage, protein percentage, lactose percentage, and ash percentage using lactoscan (Lactoscan S, software version 50, and LCD, software version 45, Nova Zagora, Bulgaria). In addition, pH values of treated milk samples were measured using pH meter (Jenway 3051 pH meter) equipped with standard combination electrodes. The apparatus was calibrated before each measure using standard buffer solutions pH 4.00 and pH 7.00 at 25°C. The pH values for each sample were recorded in comparison with fresh raw milk.

### Analysis of deltamethrin residues

#### Standard

The standard of deltamethrin (97-99%) was granted by Pak China.

#### Deltamethrin in water, feed and milk samples

Deltamethrin residues were assessed by HPLC-ultraviolet (UV) by chromatograph equipped with UV detector in water according to Hanan et al. [16], feed according to Boussahel et al. [17], and milk samples according to Darko and Acquaah [18]. Accordingly, the suitable conditions of HPLC were HPLC apparatus (Agilent1100) equipped with diode array detector; column: Zorbax SBC 18 (150 mm×4.6 mm×0.5 um film thickness); mobile phase: Acetonitrile: distilled deionized water (80:20); flow rate: 1.0 mL/min.; and detector: 226 nm UV.

The deltamethrin residues in the examined samples were compared with those obtained from similar injections of the standard solutions. Quantitative determination of these residues was obtained by the measurement of the peak areas in the chromatogram [19].

### Statistical analysis

The statistical analysis of quantitative data of deltamethrin concentration in milk samples was estimated by univariate analysis of variance (ANOVA), one-way ANOVA, and independent t-tests using SPSS program version 20 [20]. The results were considered significantly different at p<0.05. The experiment was done 3 times and the values indicated were the average of triplicate±standard error.

## Results

Deltamethrin in water and feeds existed at the 1^st^, 2^nd^, and 3^rd^ days after application of deltamethrin, and in contrary, the residues were not detected at the 7^th^, 15^th^, 21^st^, and 35^th^ days after application (Table-1).

**Table-1:** Deltamethrin residues in water and feed collected from cow’s and goat’s farms.

Samples	Deltamethrin residue	

	Water (μg/L)	Feed (μg/Kg)
Before application	0.00±0.00^a^	0.00±0.00^a^
After application		
1^st^ day	280.77±17.80^a^	381.30±26.57^a^
2^nd^ day	92.49±6.89^b^	94.29±8.30^b^
3^rd^ day	27.05±3.66^c^	41.99±4.10^c^
7^th^ day	ND*	ND
15^th^ day	ND	ND
21^st^ day	ND	ND
35^th^ day	ND	ND

* ND=Not detected. The means with different superscript in the same column indicate significant difference (p<0.05). The values indicated were the mean±standard error

**Figure-1:**
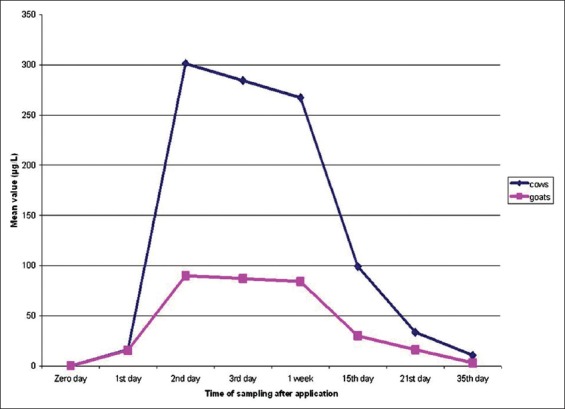
The persistence of deltamethrin (%) in cow and goat milk. Blue line referred to the persistence of deltamethrin residues in cow’s milk, but the red line referred to the persistence of deltamethrin residues in goat’s milk during the period of the study.

Deltamethrin residues in cow’s and goat’s milk samples recorded the highest value at the 2^nd^ day followed by the 3^rd^, 7^th^, and 15^th^ days, after application above MRLs, while it was detected at the 21^st^ and 35^th^ days after application in cow’s and goat’s milk within MRLs as shown in Table-2 and Figure-1.

**Table-2:** Deltamethrin residues in cow and goat milk samples.

Samples	Deltamethrin residue (μg/L)	

	Cow’s milk	Goat’s milk
Before application	0.00±0.00^d^	0.00±0.00^d^
After application		
1^st^ day	16.33±2.99^d^	15.87±1.22^c^
2^nd^ day	301.30±27.01^a^	90.00±4.22^a^
3^rd^ day	284.31±25.58^b^	87.08±3.75^a^
7^th^ day	267.32±24.06^b^	84.17±3.29^a^
15^th^ day	99.19±9.78^c^	30.01±3.82^b^
21^st^ day	33.59±5.40^d^	16.25±1.65^c^
35^th^ day	10.78±2.47^d^	3.17±3.01^d^

The means with different superscript in the same column indicate significant difference (p<0.05). The values indicated were the mean±standard error

**Figure-2:**
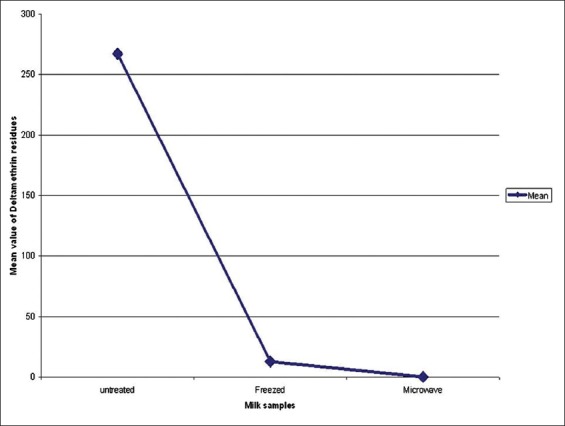
Concentration of deltamethrin residues before and after treatment of cow’s milk by freezing and microwaving (μg/L). The blue line referred to the mean value of deltamethrin residues in cow’s milk samples, and the values indicated were the mean±standard error.

Regarding the deltamethrin residues in treated polluted cow’s milk samples, the residues were not detected at the 2^nd^ day after application after exposure to microwave at 900 W for 75 s, but the mean values decline to 12.6±3.24 μg/L after freezing at −20 for 1 week (Figure-2).

Regarding the effect of both freezing and microwaving on chemical constituents of milk, microwaving did not affected chemical composition of milk in which they were nearly similar to those in fresh raw milk. However, by freezing, there was a relevant decline in the concentration of fat. It was 5.59±0.391 in compare to 7.69±0.074 in fresh raw milk (Table-3).

**Table-3:** Effect of microwaving and freezing on chemical profile of milk.

Chemical parameter (%)	Fresh raw milk (control)	Treated samples	

		Microwaving	Freezing
Fat	7.69±0.04^a^	7.68±0.021^a^	5.59±0.213^b^
SNF	6.62±0.012^a^	6.63±0.109^a^	6.56±0.580^b^
Protein	2.47±0.006^a^	2.49±0.009^b^	2.49±0.006^b^
Lactose	3.45±0.006^a^	3.47±0.007^b^	3.51±0.026^c^
Ash	0.66±0.004^a^	0.65±0.011^a^	0.63±0.014^a^
pH	6.51±0.007^a^	6.49±0.003^b^	6.72±0.0580^c^

abc Values in the same row having different superscripts differ significantly (p<0.05). The values indicated were the mean of triplicates±standard error. SNF=Solid-not-fat

## Discussion

Synthetic pyrethroids often serve as replacements for controlling pests in agriculture and animals due to their effectiveness against lice treatment and mosquito. In recent years, there is an increase in the public concern against the cancer risk related to chronic low-level exposure to pesticide residues in milk and other foods [21]. Deltamethrin residues were detected in both water and feed at different levels up to the first 3 days after its application. This may be attributed to the poor biosafety measures such as lack of precaution during pesticides spraying, absence of special area for pesticides spraying, and drinker, not frequency changed after spraying [22]. The current results clarify that deltamethrin could be not detected from the 7^th^ day in feed and water after its application. Similar results were obtained by Thomson [23].

The current results have indicated that both feed and water act as main sources of entry of pesticides into the animal body. Once the animal body system gets contaminated with pesticides residues, not only does it affect the animal directly but also exerts an indirect effect on human health through the food of animal origins such as milk and meat [24].

Deltamethrin residues were detected in all cow’s and goat’s milk samples up to the 35^th^ day after insecticides application. The highest levels were detected at the 2^nd^ day then at the 7^th^ day followed at the 15^th^ day after application as such levels were above the maximum residue limit (50 µg/kg) stated by EU pesticides database [25]. The current findings agreed with those recorded by El-Maghraby [26] who detected deltamethrin in milk samples collected for 28 days subcutaneously from treated cows with 2000 μg/kg deltamethrin. The current findings slight disagreed with those recorded by Castillo et al. [27] who recorded that the deltamethrin was rapidly absorbed and slowly excreted. Furthermore, they found that the level of deltamethrin residues in milk was very low, <1% of the treatment dose, and maximum levels were reached after 2 days 9 µg/kg for 0.1 g deltamethrin and 53 µg/kg for 1 g deltamethrin. For two concentrations, no residue level was detected after 8 days of the pour-on application.

Deltamethrin was previously detected in milk at higher concentrations during the studies performed by Misra et al. [28] and Shahzadi et al. [29]. On the other hand, Nasr et al. [30] failed to detect any pyrethroid residues in the examined samples of cow milk.

The variation in the persistence of pesticides may be attributed to the type and concentration of pesticides, the source of feed and water as well as the mode of pesticide application [31].

Deltamethrin residues were not detected in all cows’ milk samples which had the highest concentration at the 2^nd^ day after treatment of the milk by microwave, but there were 12.6±3.24 μg/L after treatment of the milk by freezing; this indicated that the microwaving of milk samples is more effective than the freezing for destroying of deltamethrin residues in milk samples.

There is no doubt that milk is excellent nutrition for the human being due to its content of proteins, fat, lactose, minerals, and vitamins. In addition, apart from that, milk proteins can also exert numerous antimicrobial and physiological activities benefiting the consumer in a variety of ways. These activities include enhancement of immune function and defending against pathogenic bacteria, viruses, and yeasts [32].

Regarding the effect of both freezing and microwaving on the milk chemical constituents, microwaving was shown to be devoid from any effect on the chemical composition of milk, being the same as to those of fresh raw milk. However, by freezing, the concentration of fat as compared to that of fresh raw milk was significantly declined. This effect was similar to that obtained by Garcı´a-Lara et al. [33] and Vieira et al. [34]. Freezing of milk gives rise to a series of physical changes in its fat content such as rupture of the fat globule membranes and alteration of casein micelles [33].

In the same context, Oliveira et al. [35] reported that at the temperature of −20°C, lipase activity is maintained, and therefore, there is active lipolysis. This breaks down the triglycerides, reduces their content, and increases the monoglycerides, diglycerides, and free fatty acids content.

At the same time, the levels of deltamethrin in milk samples were very lower than the recommended MRL [25] after milk treatment by microwaving or freezing without affecting milk composition.

The deltamethrin is stable at 40°C in the dark, at room temperature in the light, and its melting point is 98-101°C. Microwave treatment of milk at 900 W for 75 s can destroy the deltamethrin residues in milk. This can be attributed to the fact that the high temperature can reduce the amount or the value of the pesticide residue [36]. The estimated pesticide residues in microwaved milk samples showed the efficient role of heat processing perhaps due to evaporation, codistillation, and thermal degradation which vary with the chemical nature of pesticides [37].

Microwaved milk sample content of residues was below the detectable levels in raw milk. These results are in agreement with that reported by Abou-Arab[38], who showed the efficient role of heat treatment on the degradation of some pesticides in milk products.

The results of this work suggested that the consumption of heat-treated milk and milk products may be safer than consumption of raw milk also indicated that some of the pyrethroid pesticides still contaminate the environment resulting in contamination of foodstuffs, particularly milk. There is a potential risk of the consumption of such contaminated milk on human’s health, particularly infants and children [39-42].

Milk contamination with the pesticides residues can be controlled by preventing the contamination of feedstuffs. The findings of the study might help in extending awareness in dairy farmers and local people about pesticides and their hazardous effects on human.

## Conclusion

Feed and water act as important sources of pesticides into animal body, also deltamethrin does not persist in the environment. It disappears in both feed and water within 1 week, so it considered biodegradable and save insecticides. Moreover, our results concluded that microwave is an effective method for the elimination of deltamethrin from milk than freezing.

It can be concluded from the analysis of milk samples that treated by microwave play an important role in reducing the concentration of deltamethrin in milk without affecting its chemical profile.

## Authors’ Contribution

HEKE, HAE, and SSI designed the concept for this research and scientific paper. HEKE and SSI have conducted the maintenance of dairy animals used in an experiment in the farm, collecting samples, and compiling the resource materials. HAE was provided technical supports, made chemical examination of milk samples, and analyzed data. HEKE applied the samples for HPLC. All authors participated in manuscript’s draft and revision. All authors have read and approved the final manuscript.

## References

[1] Elliott M, Farnham A.W, Janes N.F, Needhan D.H, Pulman D.A (1974). Synthetic insecticides with a new order of activity. Nature.

[2] Côté J, Bonvalot Y, Carrier G, Lapointe C, Fuhr U, Tomalik-Scharte D (2014). A novel toxicokinetic modeling of cypermethrin and permethrin and their metabolites in humans for dose reconstruction from biomarker data. PLoS One.

[3] Frank B.A, Gadi V.P (2015). Toxicological effects of pyrethroids on non-target aquatic insects. Environ. Toxicol. Pharmacol.

[4] Ray D.E, Forshaw P.J (2000). Pyrethroid insecticides:Poisoning syndromes, synergies and therapy. J. Toxicol. Clin. Toxicol.

[5] Hasibur R, Al-Thbiani A, Shalini S, Zahid K.A, Anand M, Abid A.A (2014). Systematic review on pyrethroid toxicity with special reference to deltamethrin. J. Entomol. Zool. Stud.

[6] Páleníková A.G, Martínez-Domínguez F.J, Arrebola R, Romero-González A.G (2015). Determination of pesticides and transformation products in*Ginkgo biloba*nutraceutical products by chromatographic techniques coupled to mass spectrometry. Food Anal. Methods.

[7] Koesukwiwat U, Jayanta S, Leepipatpiboon N (2007). Validation of a liquid chromatography-mass spectrometry multi-residue method for the simultaneous determination of sulfonamides, tetracyclines, and pyrimethamine in milk. J. Chromatogr. A.

[8] Kolberg D.I.S, Presta M.A, Wickert C, Adaime M.B, Zanelli R (2009). Rapid and accurate simultaneous determination of abamectin and ivermectin in bovine milk by high performance liquid chromatography with fluorescence. J. Braz. Chem. Soc.

[9] Bagg R, Vessie G.H, Dick C.P, Duffield T, Wilson J.B, Aramini J.J (2005). Milk residues and performance of lactating dairy cows administered high doses of monensin. Can. J. Vet. Res.

[10] Tsiplakou E, Anagnostopoulos C.J, Liapis K, Haroutounian S.A, Zervas G (2010). Pesticides residues in milk and feedstuff of farm animals drawn from Greece. Chemosphere.

[11] Danaher M.L, Howell C.S, Crooks R.C, Flajs V.C, Keeffe M. O (2006). Review of methodology for the determination of macrocyclic lactone residues in biological matrices. J. Chromatogr. B.

[12] Cerkvenik-Flajs V.L, Milčinski L.A.H, Süssinger M.D, Antonić J (2010). Trace analysis of endectocides in milk by high performance liquid chromatography with fluorescence detection. Anal. Chim. Acta.

[13] David W, Arno J, Jacob H, Michael L, Peter P.C, Steve H (2013). Development and validation of a 'universal'HPLC method for pyrethroid quantification in long-lasting insecticidal mosquito nets for malaria control and prevent. Trop. Med.Int. Health.

[14] Tremonte P, Luca T, Mariantonietta S, Gianfranco P, Luisa F, Valeria C, Raffaele C, Elena S (2014). Raw milk from vending machines:Effects of boiling, microwave treatment, and refrigeration on microbiological quality. J. Dairy Sci.

[15] Tomer V, Sangha J.K (2013). Vegetable processing at household level:Effective tool against pesticide residue exposure. IOSR J. Environ. Sci. Toxicol. Food Technol.

[16] Hanan A.A, Fayza A.S, Fayza A, El Tedawy A, Sallam A (2016). Detection of ivermectin and deltamethrin in the bulk milk tank of some dairy herd. Adv. Environ. Biol.

[17] Boussahel R, Moussaoui K.M, Harik D (2006). Determination of residues of deltamethrin in wheat and potato by HPLC. Afr. J. Agric. Res.

[18] Darko G, Acquaah S.O (2008). Level of organochlorine pesticides residues in dairy products in Kumasi, Ghana. Chemosphere.

[19] Tentu N.R, Patrudu T.B, Babu K.R, Sreenivasul E.G, Karri A (2014). A novel method for determination of deltamethrin residues in aquatic tox medium followed by gas chromatography mass spectrometry method. Int. J. Pure Appl. Sci. Technol.

[20] Amro S.M, Omima M.E, Osama H.M (2014). Effect of effective microorganisms (EM) and potassium sulphate on productivity and fruit quality of “Hayany” Date palm grown under salinity stress. IOSR J. Am. Chem. Soc.

[21] Kim S.S, Lee R.D, Lim K.J, Kwack S.J, Rhee G.S, Seok J.H, Lee G.S, Jeung E.B, Park K.L (2005). Potential estrogenic and antiandrogenic effects of permethrin in rats. J. Reprod. Dev.

[22] Goodarzi M, Ortiz E.V, Coelho L.S, Duchowicz P.R (2010). Linear and non-linear relationships mapping the Henry's law parameters of organic pesticides. Atmos Environ.

[23] Thomson W.T (1989). Agricultural Chemicals. Book I:Insecticides.

[24] Stefanelli P, Santilio A, Cataldi L, Dommarco R (2009). Multiresidue analysis of organochlorine and pyrethroid pesticides in ground beef meat by gas chromatography-mass spectrometry. J. Environ. Sci. Health, Part B.

[25] EU Pesticides Database MRLs for Commission Regulation (EU) 2015/1200 of 22 July (2015). Amending Annexes II and III to Regulation (EC) No 396/2005 of the European Parliament and of the Council as regards maximum residue levels for Pesticides.

[26] El-Maghraby S (2007). Metabolism of deltamethrin in rats. Biomed. Environ. Sci.

[27] Castillo A.R, St-Pierre N.R, Silva N, Weiss W.P (2013). Mineral concentrations in diets, water, and milk and their value in estimating on-farm excretion of manure minerals in lactating dairy cows. J. Dairy Sci.

[28] Misra U, Singh S.P, Ahmad A.H, Hore S.K, Sharma L.D (2005). Synthetic pyrethroid residues in foods of animal origin in Kumaon. Toxicol. Int.

[29] Shahzadi N, Imran M, Sarwar M, Hashmi A.S, Wasim M (2013). Identification of pesticides residues in different samples of milk. J. Agro Alim. Proc. Technol.

[30] Nasr I.N, Sallam A.A.A, Abd El-Khair A.A (2007). Monitoring of certain pesticide residues and some heavy metals in fresh cow's milk at Gharbia Governorate, Egypt. J. Appl. Sci.

[31] Turi M.S, Sods K, Vegh E (2008). Determination of residues of pyrethroid and organophosphorus ectoparasiticides in foods of animal origin. Acta. Vet. Hung.

[32] López-Expósito I, Recio I (2008). Protective effect of milk peptides:antibacterial and antitumor properties. Adv. Exp. Med. Biol.

[33] Garcı´a-Lara N.R, Escuder-Vieco D, Garcı´a-Algar O, De la Cruz J, Lora D, Palla´S-Alonso C (2012). Effect of freezing time on macronutrients and energy content of breast milk. Breast Feed. Med.

[34] Vieira A.A, Soares F.V, Pimenta H.P, Abranches A.D, Moreira M.E (2011). Analysis of the influence of pasteurization, freezing/thawing, and offer processes on human milk's macronutrient concentrations. Early Hum. Dev.

[35] Oliveira S.C, Amélie D, Olivia M, Amandine B, Florence R, Gwénaële H, Emelyne D, Frédéric C, Didier D, Claire B (2016). Holder pasteurization impacts the proteolysis, lipolysis and disintegration of human milk under*in vitro*dynamic term newborn digestion. Food Res. Int.

[36] Eldakroory S.A, El Morsi D.A, Abdel-Rahman R. H, Roshdy S, Gouida M. S, Khashaba E.O (2017). Correlation between toxic organochlorine pesticides and breast cancer. Hum. Exp. Toxicol.

[37] Sharma J.S, Satya V, Kumar D.K, Tewary D.K (2005). Dissipation of pesticides during breadmaking. Chem. Health Saf.

[38] Abou-Arab A.K (1999). Effects of processing and storage of dairy products on lindane residues and metabolites. Food Chem.

[39] FAO/WHO (2008). Food Standards Program. Codex Alimentarius. Pesticide Residues in Food.

[40] Faqir M, Awais M.M, Akhtar M, Anwa M.I (2013). Quantitative Structure activity relationship and risk analysis of some pesticides in the cattle milk. Iran. J. Environ. Health Sci. Eng.

[41] POP's Office (2006). Polychlorinated Biphenyls PCB's-reduction and elimination.

[42] WHO (2003). World Health Organization. Persistent Organic Pollutants Compiled by the Program for the Promotion of Chemical Safety, The Division of Control of Tropical Diseases, and the Food Safety Unit.

